# An organotrophic *Sideroxydans* reveals potential iron oxidation marker genes

**DOI:** 10.1128/aem.00395-25

**Published:** 2025-08-14

**Authors:** Rene L. Hoover, Kirsten Küsel, Clara S. Chan

**Affiliations:** 1 Microbiology Graduate Program, University of Delaware 5972, Newark, Delaware, USA; 2 Department of Earth Sciences, University of Delaware 5972, Newark, Delaware, USA; 3 Aquatic Geomicrobiology, Institute of Biodiversity, Friedrich Schiller University Jena 9378, Jena, Germany; 4 Cluster of Excellence Balance of the Microverse, Friedrich Schiller University, Jena, Germany; 5 Delaware Biotechnology Institute, Newark, Delaware, USA; Washington University in St. Louis 7548, St. Louis, Missouri, USA

**Keywords:** iron oxidation, *Sideroxydans*, Gallionellaceae, iron-oxidizing bacteria, mixotrophy, *cyc2*, *ircABCD*

## Abstract

To understand the ecophysiology and the role of iron-oxidizing bacteria (FeOB) in various ecosystems, we need to identify marker genes of the iron oxidation pathway to track activity *in situ*. The Gallionellaceae *Sideroxydans* sp. CL21, an autotrophic iron-oxidizing bacterium isolated from a peatland, is unusual among FeOB isolates in its genomic potential to utilize organic compounds as energy sources. Therefore, it offers the unique opportunity to determine genes expressed under litho- vs organotrophic conditions. We demonstrated the growth of *Sideroxydans* sp. CL21 on organic substrates (lactate and pyruvate) and inorganic substrates [Fe(II), magnetite, thiosulfate, and S(0)]. Thus, cells were capable of lithoautotrophic, organotrophic, and potentially organoheterotrophic growth. Surprisingly, when lactate-grown cells were given Fe(II), mid-log phase cells were unable to oxidize iron, while late-log phase cells oxidized iron. To elucidate iron oxidation pathways, we compared gene expression between mid-log (non-iron-oxidizing) and late-log (iron-oxidizing) lactate-grown cells. Genes for iron oxidases (*cyc2*, *mtoA*) were highly expressed at both time points but did not correspond to iron oxidation capability, making them unsuitable marker genes of iron oxidation activity by themselves. However, genes encoding periplasmic and inner membrane cytochromes were significantly upregulated in cells capable of iron oxidation. These genes include *mtoD*, *cymA/imoA*, and a cluster of Fe(II)-responsive genes (*ircABCD*). These findings suggest that Gallionellaceae regulate their iron oxidation pathways in multiple stages, with iron oxidase-encoding genes proactively expressed. Other genes encoding electron carriers are upregulated only when iron oxidation is needed, which makes these genes (i.e., *ircABCD*) good prospective indicators of iron oxidation ability.

**IMPORTANCE:**

Iron-oxidizing bacteria (FeOB) are widespread in the environment, and we suspect that they play key roles in nutrient and other elemental cycles. However, with no isotopic marker, we lack the ability to monitor FeOB activity, prompting us to search for genetic markers. Previous work suggests that expression of iron oxidase genes does not directly correspond to iron oxidation activity in Gallionellaceae, and little was known about the other genes in the pathway. Here, we study a unique FeOB isolate that possesses organotrophic capabilities and demonstrate its potential for mixotrophic growth on lactate and Fe(II). Its ability to oxidize iron is regulated, allowing us to discover potential iron oxidation pathway genes with expression that corresponds to iron oxidation activity. If these genes can be further validated as iron oxidation marker genes, they will enable us to delineate autotrophic and organoheterotrophic FeOB impacts on carbon cycling in wetlands and other natural and engineered environments.

## INTRODUCTION

Iron oxidation is a widespread process in the environment, playing key roles in water and soil health as Fe(III) oxyhydroxides can adsorb various nutrients and metals (1). Despite the pervasiveness and importance of iron oxidation, there remains much uncertainty as to how much of it is abiotic vs biotic, i.e., catalyzed by iron-oxidizing bacteria (FeOB). This distinction (biotic vs abiotic) is key to understanding how iron oxidation affects other nutrient cycles, which requires (i) a deeper understanding of the C metabolisms of FeOB and (ii) a method to link microbial iron oxidation activity in the environment to C and other metabolic transformations. Although FeOB are widely thought to be primarily autotrophic, accumulating evidence suggests that a considerable number of FeOB may be mixotrophic (2–6) or organo(hetero)trophic (7, 8), expanding their potential effects on C cycling. Because these FeOB can use alternate energy sources (9–12), they are not always oxidizing iron, so we require a method of detecting active iron oxidation. To link microbial iron oxidation to biogeochemical effects, we need gene markers of iron oxidation activity.

Studying facultative FeOB allows us to discern genes specific to iron oxidation. An isolate of the Gallionellaceae, *Sideroxydans* sp. CL21, presents a new opportunity to learn about FeOB metabolism, iron oxidation mechanisms, and potential marker genes. *Sideroxydans* sp. CL21 is rare among FeOB isolates in possessing genes for organoheterotrophy, notably lactate permease and dehydrogenase. It was isolated from the Schlöppnerbrunnen fen (13), a semi-acidic (pH 4.5–5.5), iron-rich peatland in Northern Germany (14–16). *Sideroxydans* sp. CL21 is closely related to the well-studied *Sideroxydans lithotrophicus* ES-1 (9) but differs from ES-1 in that its genome is ~20% larger and encodes genes for organoheterotrophy (17, 18). *Sideroxydans* sp. CL21 has been shown to grow on the non-iron substrates H_2_ and thiosulfate (18). It has also been grown on the reduced iron substrates FeS and Fe(0) (13, 18), but neither of these is a pure Fe(II) substrate as Fe(0) evolves H_2_, so growth on Fe(II) alone has not been demonstrated. *Sideroxydans* sp. CL21 has also been grown in the presence of mixed substrates, including lactate/FeS and lactate/Fe(0) (13, 18). However, previous cultures did not grow on lactate alone (13, 18). Thus, although the growth in the presence of combined organic and inorganic electron donors suggests a potential for mixotrophy, further work is needed to clarify exactly which substrates promote *Sideroxydans* sp. CL21 growth and support organoheterotrophy.

Markers of iron oxidation activity could be the genes within iron oxidation pathways, but our knowledge of these pathways is still in development. The *Sideroxydans* sp. CL21 genome encodes several genes related to iron oxidation and associated pathways. Its genome encodes two known iron oxidases: (i) the monoheme cytochrome-porin Cyc2 and (ii) the decaheme cytochrome MtoA and associated porin MtoB (17–19). In fact, the genome has three copies of *cyc2* and an *mtoAB*, plus an additional copy of *mtoA* next to an unnamed porin. While the presence of *cyc2* or *mtoA* indicates iron oxidation capability, it is unclear if they are suitable genetic markers of iron oxidation *activity*. Ideally, a gene marker would exhibit little to no expression when there is no iron oxidation activity and be upregulated during iron oxidation, with expression level scaling with activity. Yet, in the closely related *S. lithotrophicus* ES-1, one copy of *cyc2* is highly expressed in both iron-oxidizing and thiosulfate-oxidizing cells, while the other two copies have low expression during thiosulfate oxidation but are upregulated when oxidizing iron (10). In marine hydrothermal environments, the *cyc2* gene is highly expressed by various iron-oxidizing Zetaproteobacteria, and expression increases in microcosms amended with Fe(II) (20). In the facultative FeOB Zetaproteobacteria *Ghiorsea bivora* TAG-1, *cyc2*_TAG-1_1_ and Cyc2_TAG-1_1_ are expressed highly under both H_2_- and iron-oxidizing conditions (21). Together, this suggests that *cyc2*/Cyc2 expression may not necessarily correspond to iron oxidation activity. There is less information on *mtoAB/*MtoAB, though these iron oxidase genes/proteins are upregulated in *S. lithotrophicus* ES-1 grown on Fe(II) minerals (22, 23). *Sideroxydans* sp. CL21 also encodes two gene clusters, predicted to encode porin-cytochrome complexes (PCC), each with two multiheme cytochromes (PCC3_copy1_, cytochromes have 24 and 14 hemes; PCC3_copy2_, cytochromes have 24 and 12 hemes). PCC3 is hypothesized to also play a role in extracellular electron transport and possibly iron oxidation (24). Additional putative components of the iron oxidation pathway (*mtoD*, *cymA/imoA*, and Slit_1321-1324) are less well studied; these include genes for inner membrane and periplasmic electron carriers. In all, *Sideroxydans* sp. CL21 has a wide variety of genes that are known or predicted to participate in iron oxidation, giving multiple possibilities for marker genes.

Here, we constrain potential iron oxidation marker genes by studying differential gene expression in *Sideroxydans* sp. CL21. We first optimized the cultivation conditions by testing *Sideroxydans* sp. CL21’s growth on organic acids alone, plus other Fe(II) and non-iron substrates. Cells grew quickly and to the highest cell density on organic acids. We then tested the ability of these organic-grown cells to oxidize iron and found that lactate-grown cells exhibited a difference in iron-oxidizing phenotype between mid-log (no iron oxidation activity) and late-log (iron oxidation activity). This enabled us to compare gene expression between iron-oxidizing and non-iron-oxidizing conditions to detect genes whose expression corresponds to iron oxidation potential. The genes identified help expand our knowledge of the iron oxidation pathway and provide a list of candidate marker genes for iron oxidation.

## RESULTS

### Physiology and growth on Fe(II), S, and organic substrates

**Fig 1:**
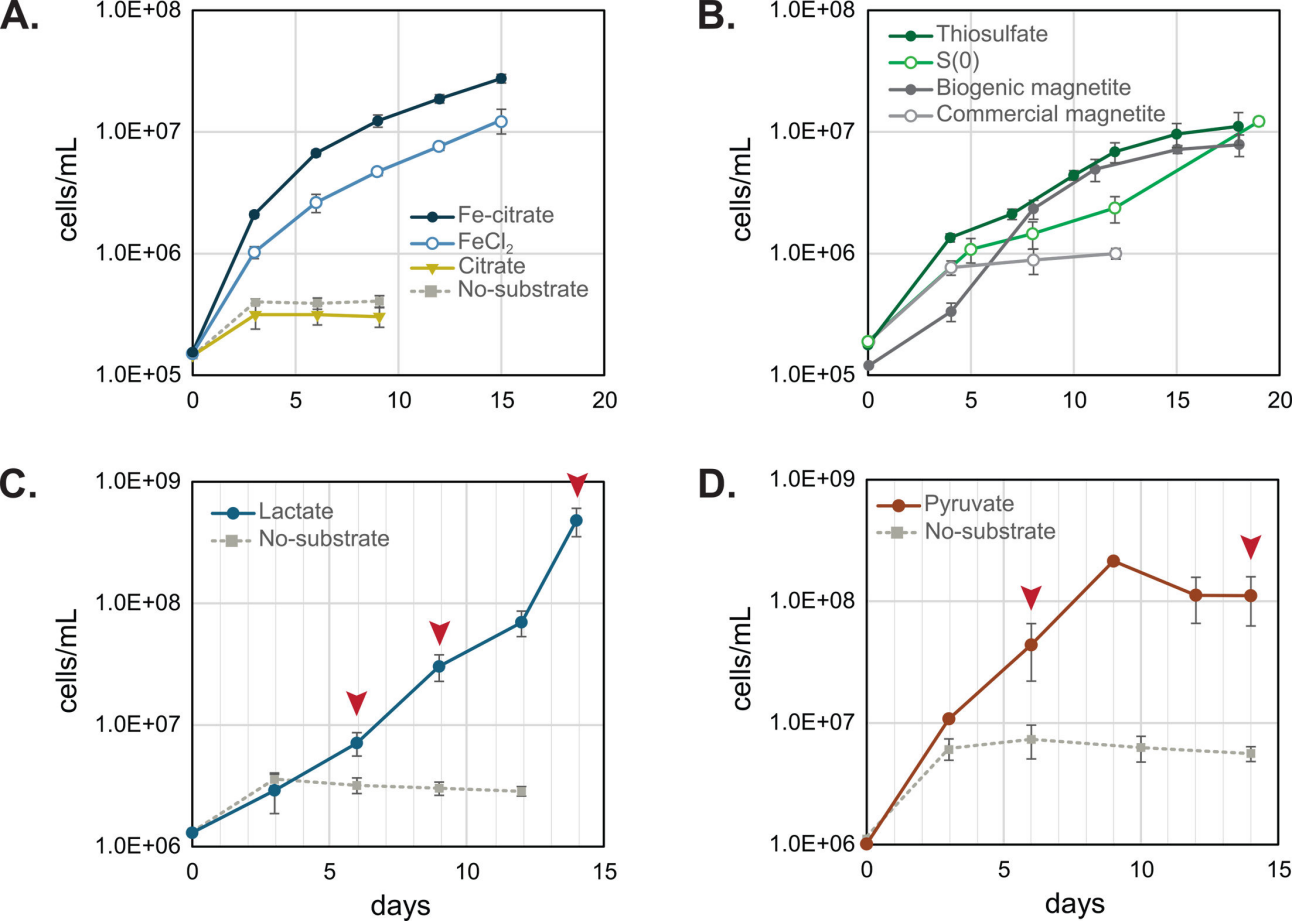
Growth of *Sideroxydans* sp. CL21 on (**A**) FeCl_2_, FeCl_2_ + citrate, and citrate compared to a no substrate control; (**B**) thiosulfate, S(0), and biogenic and commercial magnetite; (**C**) lactate; and (**D**) pyruvate. Red arrows in panel C indicate days cells were sampled for the Fe(II) spike experiments and transcriptome sequencing. Red arrows in panel D indicate days cells were sampled for Fe(II) spike experiments. Error bars represent one SD between three replicates.

*Sideroxydans* sp. CL21 grew on a range of individual organic and inorganic electron donors (Fig. 1). *Sideroxydans* sp. CL21 grew well on dissolved, ferrous iron (FeCl_2_), reaching a cell density of 1.25 × 10^7^ cells/mL in 15 days (Fig. 1A). This growth on FeCl_2_ was improved when media included 2.5 mM sodium citrate to complex iron, reaching a density of 2.76 × 10^7^ cells/mL (Fig. 1A). Attempts to culture *Sideroxydans* sp. CL21 on 5 mM sodium citrate alone showed no growth beyond that of a no-substrate control (Fig. 1A), demonstrating *Sideroxydans* sp. CL21 cannot conserve energy from citrate. As Fe(II) was the only available electron donor, these results confirm that *Sideroxydans* sp. CL21 can grow as a lithoautotrophic iron oxidizer.

*Sideroxydans* sp. CL21 was cultured on commercial and biogenic magnetite to assess its growth on solid iron minerals. Cells grew better on the biogenic vs commercial magnetite (Fig. 1B). This may be due to the lower crystallinity and higher solubility of biogenic magnetite, which sheds more Fe(II) into solution than commercial magnetite (25). The biogenic magnetite, which was produced from ferrihydrite by the iron-reducer *Shewanella*, may also contain extracellular metabolites that could enhance iron oxidation activity and growth in *Sideroxydans* sp. CL21 (26).

*Sideroxydans* sp. CL21 was also tested for growth on sulfur substrates. The genome includes *dsrAB* genes that encode a reverse dissimilatory sulfite reductase (19). It has previously been cultured on FeS and in agarose-stabilized media in gradient tubes with either thiosulfate or thiosulfate plus H_2_ or lactate (18). Since agarose may provide a source of C (27, 28), we used liquid media without an organic carbon source to confirm whether cells could grow using individual sulfur substates as their sole electron donor. In these experiments, *Sideroxydans* sp. CL21 grew on thiosulfate and elemental sulfur (Fig. 1B), reaching densities near 10^7^ cells/mL. However, growth was slower (18–19 days), and cell density was lower than that of Fe(II)-grown cells (Fig. 1A and B). Nonetheless, this shows *Sideroxydans* sp. CL21 is capable of two additional lithoautotrophic metabolisms using thiosulfate and S(0) as electron donors.

**Fig 2:**
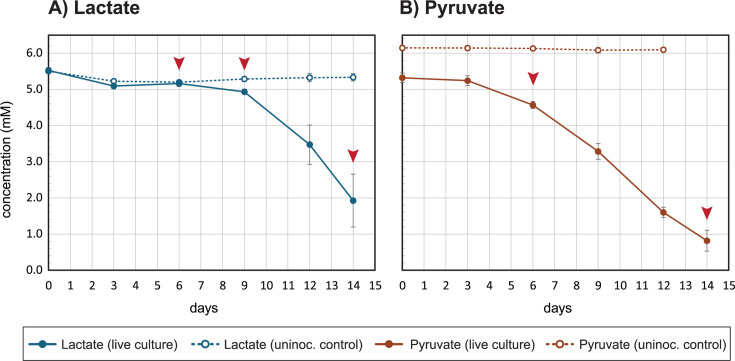
Consumption of (**A**) lactate and (**B**) pyruvate over time by live *Sideroxydans* sp. CL21 in growth experiments (Fig. 1C and D) vs uninoculated controls. Arrows indicate sampling points for short-term iron oxidation assays (Fig. 3). Error bars represent one SD between three replicates.

Previous studies showed that the growth of *Sideroxydans* sp. CL21 in multi-substrate experiments where lactate was combined with inorganic substrates [FeS, Fe(0), H_2_, and NaS_2_O_3_] in agarose gradient tubes (18), yet cells were never successfully grown on lactate alone using these methods. Our data show that *Sideroxydans* sp. CL21 grew in liquid culture with either 5 mM lactate (Fig. 1C and 2A) or 5 mM pyruvate (Fig. 1D and 2B) as the sole electron donor. It is unclear why growth on lactate was positive in our study but previously negative, but it could be due to the more controlled oxygen concentrations in the headspace of these serum bottle liquid cultures vs a gradient tube. Cells cultured on lactate grew to an order of magnitude higher density than those cultured on Fe(II), reaching 4.8 × 10^8^ cells/mL in 14 days (Fig. 1C). Together, these results show that *Sideroxydans* sp. CL21 can grow organotrophically, and likely organoheterotrophically, on lactate or pyruvate.

### The potential for mixotrophic energy metabolism

**Fig 3:**
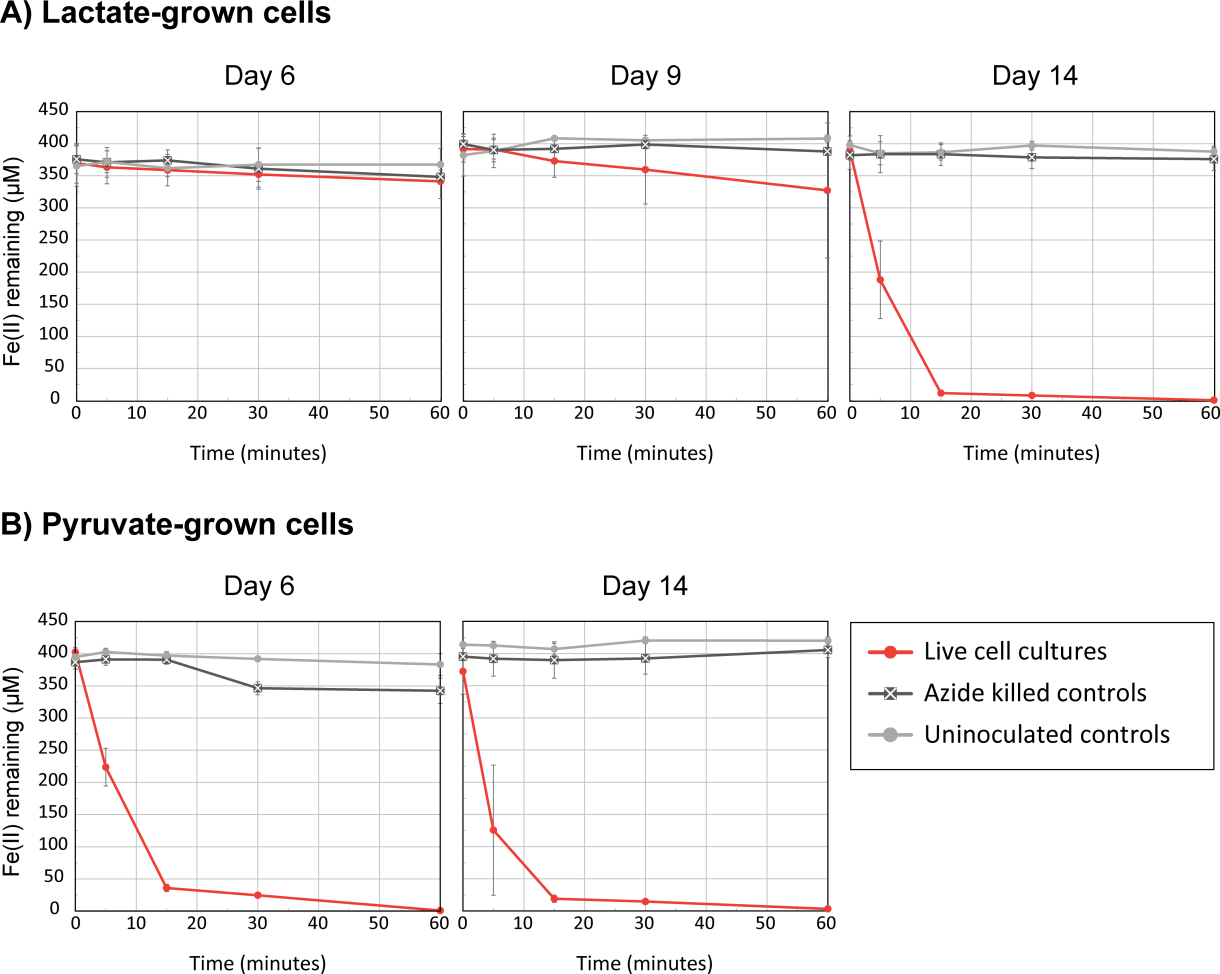
Results of Fe(II) spike assays for lactate- and pyruvate-grown cells. (**A**) Aliquots of live, lactate-grown *Sideroxydans* sp. CL21 from the transcriptome experiment (Fig. 1C and 2A) rapidly oxidized iron on day 14 compared to day 6 or day 9. (**B**) Cells from pyruvate growth experiments (Fig. 1D and 2B) rapidly oxidized iron on both day 6 and day 14. Error bars represent one SD between three replicates.

*Sideroxydans* sp. CL21 cells have the genomic potential to carry out multiple energy metabolisms simultaneously (mixotrophy), but this requires further experimental proof. Our approach was to determine whether *Sideroxydans* sp. CL21 cells grown on short-chain organic acids could also oxidize ferrous iron in a short-term assay. We first harvested lactate-grown cells on days 6, 9, and 14, then added a 400 µM “spike” of FeCl_2_, and measured Fe^2+^ concentration over time. On day 6, live cells (7.1 × 10^6^ cells/mL) did not oxidize iron within the 60-minute experiment. On day 9 (3.3 × 10^7^ cells/mL), the modest amount of iron oxidation was mostly driven by a single bottle, 6A, which had a higher cell density than the other replicates, making it closer to late-log phase (Fig. 3A, Fig. S1). In contrast, with live cells (4.8 × 10^8^ cells/mL) harvested on day 14, iron was completely oxidized within 15 minutes (Fig. 3A) despite the presence of >1 mM of lactate remaining in the cultures (Fig. 2A). This difference in response between mid- and late-log cultures indicates iron oxidation in *Sideroxydans* sp. CL21 is regulated. We also tested pyruvate-grown cells (Fig. 1D and 2B) and found that *Sideroxydans* sp. CL21 was primed to oxidize iron on both day 6 (4.4 × 10^7^ cells/mL) and day 14 (1.1 × 10^8^ cells/mL; Fig. 3B). Together, these results demonstrate that *Sideroxydans* sp. CL21 has the ability to oxidize iron while growing on organic acids, indicating that cells are capable of mixotrophic energy metabolism. Furthermore, because iron oxidation is regulated, we are able to use these samples to investigate gene expression differences between iron-oxidizing and non-iron-oxidizing conditions.

### Transcriptomics

Lactate-grown cultures were harvested for transcriptomics after aliquots were taken for the Fe(II)-spike experiments on days 6, 9, and 14 (Fig. 1C and 2A). We present the transcriptome data as *Z*-scores, calculated from the normalized, regularized log (rlog) transformed read data. This gives a measure of a gene’s expression on a log scale relative to the mean expression (*Z*-score = 0) of all genes at a given time point.

#### Expression of lactate and carbon fixation genes  

**Fig 4:**
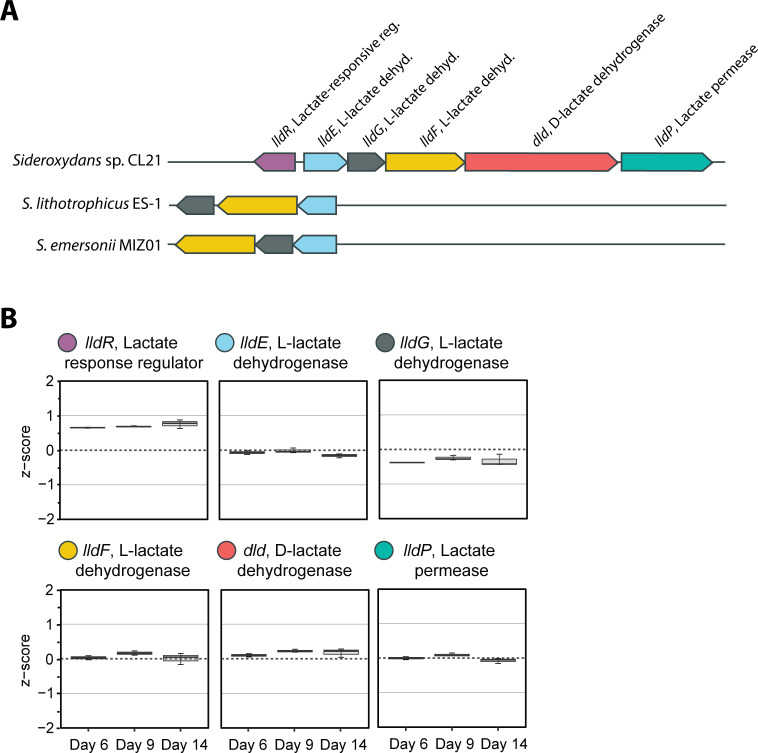
**(A**) The six lactate oxidation genes encoded by *Sideroxydans* sp. CL21 and (B) their patterns of expression on days 6, 9, and 14. Expression is represented by *Z*-score, which shows the expression of each gene relative to the mean gene expression (0) at the time point. Error bars represent the spread of the interquartile range.

The *Sideroxydans* sp. CL21 genome contains six genes for lactate oxidation. These genes are co-located in the genome and encode a lactate response regulator (LldR), L-lactate dehydrogenase (LldE), L-lactate dehydrogenase (LldG), L-lactate dehydrogenase (LldF), D-lactate dehydrogenase (Dld), and lactate permease (LldP; Fig. 4A). The genes *lldR*, *dld*, and *lldP* set *Sideroxydans* sp. CL21 apart from other closely related Gallionellaceae isolates *S. lithotrophicus ES-1* and *Sideroxyarcus emersonii* MIZ01 that are unable to grow by oxidizing lactate (Fig. 4A). As expected, all six of the *Sideroxydans* sp. CL21 lactate oxidation genes were consistently expressed throughout the experiment (Fig. 4B, Table S1). None of the genes were significantly up- or downregulated between the timepoints (Fig. 4B, Table S1), which fits with the consistent availability of lactate in the media (Fig. 2A).

When cultured solely with inorganic substrates, *Sideroxydans* sp. CL21 grows lithoautotrophically, fixing CO_2_ via ribulose bisphosphate carboxylase (RuBisCo) and the Calvin-Benson-Bassham cycle (26). High concentrations of organic carbon might be expected to promote heterotrophy and suppress autotrophy, yet lactate-grown *Sideroxydans* sp. CL21 still expressed genes for both Form I and Form II RuBisCo at all time points (Fig. S1B). Expression of Form I RuBisCo was relatively low, while expression of Form II RuBisCo was high and greater than the mean total expression at all three time points (Fig. S1B). Form II RuBisCo has a higher affinity for oxygen (29), so its higher expression relative to Form I would be consistent with the micro-oxic culturing conditions used in our experiments. While further metabolic evidence is needed, high expression of the gene encoding Form II RuBisCo suggests *Sideroxydans* sp. CL21 could continue to fix CO_2_ while oxidizing lactate.

#### Expression of iron oxidation genes

**Fig 5:**
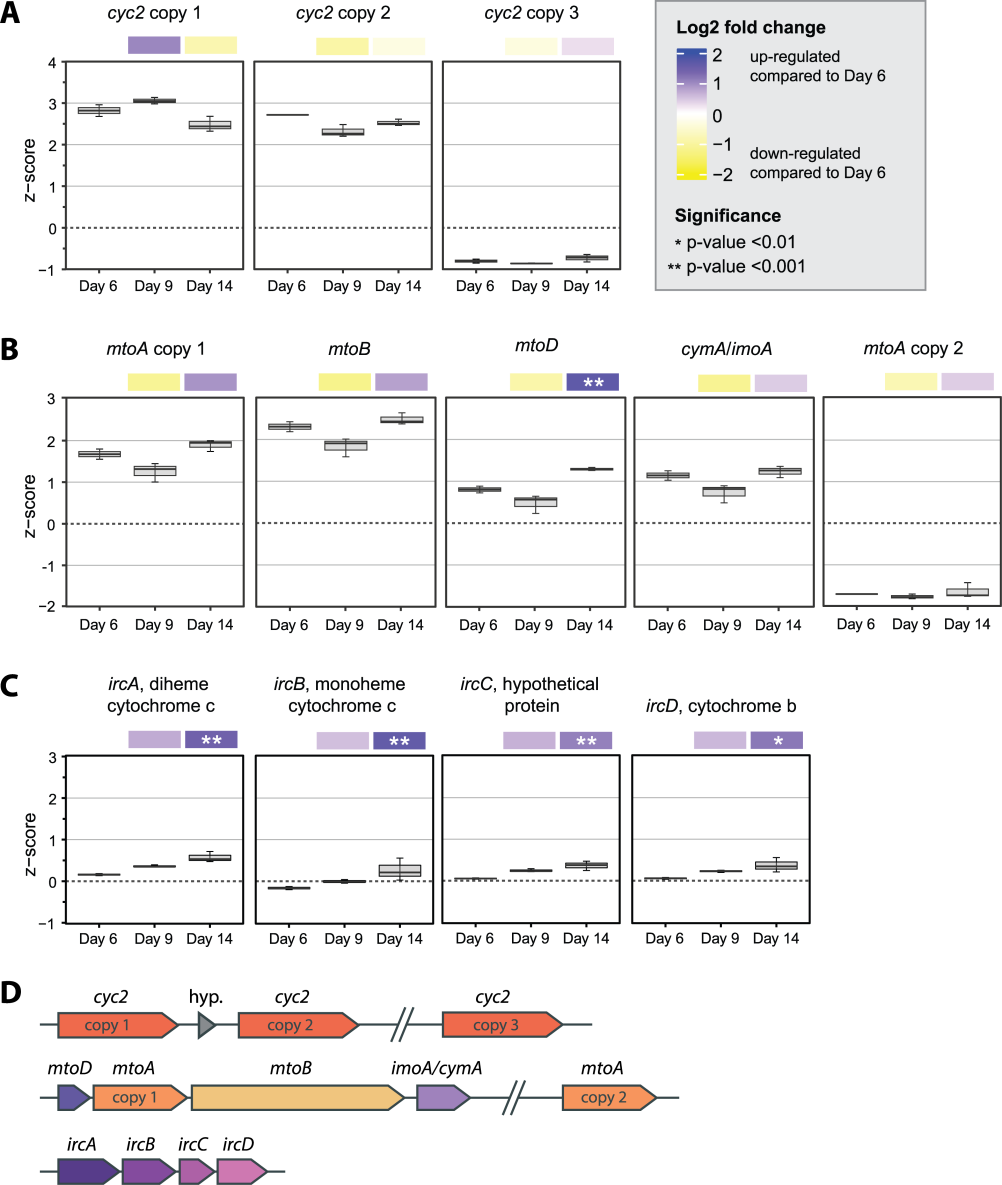
Boxplots of *Z*-score normalized expression for each time point with heatmaps showing the Log2 Fold Change in expression between day 9 and day 14 vs day 6. Expression in the boxplots is represented by *Z*-score, which shows the expression of each gene relative to the mean gene expression (0) at the time point. *Z*-score was calculated from rlog normalized count data from DESeq2. Heatmaps above the boxplots represent the Log2 Fold Change in average gene expression between day 9 and day 14 vs day 6, with statistically significant *P*-values noted with asterisks (*). Data are shown for (**A**) *cyc2* iron oxidase genes; (**B**) *mtoA* iron oxidase, *mtoB* porin, and *mtoD*, *imoA/cymA* periplasmic *c*-type cytochrome genes; and (**C**) *ircABCD* Fe(II)-responsive genes. Error bars represent the spread of the interquartile range. A gene map (**D**) shows the co-location of genes in the CL21 genome. Slashes breaking the line indicate that genes are distant, and “hyp.” denotes a gene encoding a hypothetical protein.

The different iron oxidation activities of lactate cultures prompted us to investigate whether this ability corresponds to the expression of iron oxidation genes. *Sideroxydans* sp. CL21 has multiple iron oxidase genes, including three copies of *cyc2* and two copies of *mtoA*. Though there was variation in expression between the copies, overall, both *cyc2* and *mtoA* were highly expressed on all 3 days (Fig. 5A and B). Compared to day 6 (no iron oxidation), expression of *cyc2* or *mtoA* genes was not statistically significantly up- or downregulated on day 14 when iron oxidation was rapid (Fig. 5A and B, Table S1). Thus, expression of known iron oxidase genes did not correspond to the cells’ ability to oxidize iron.

**Fig 6:**
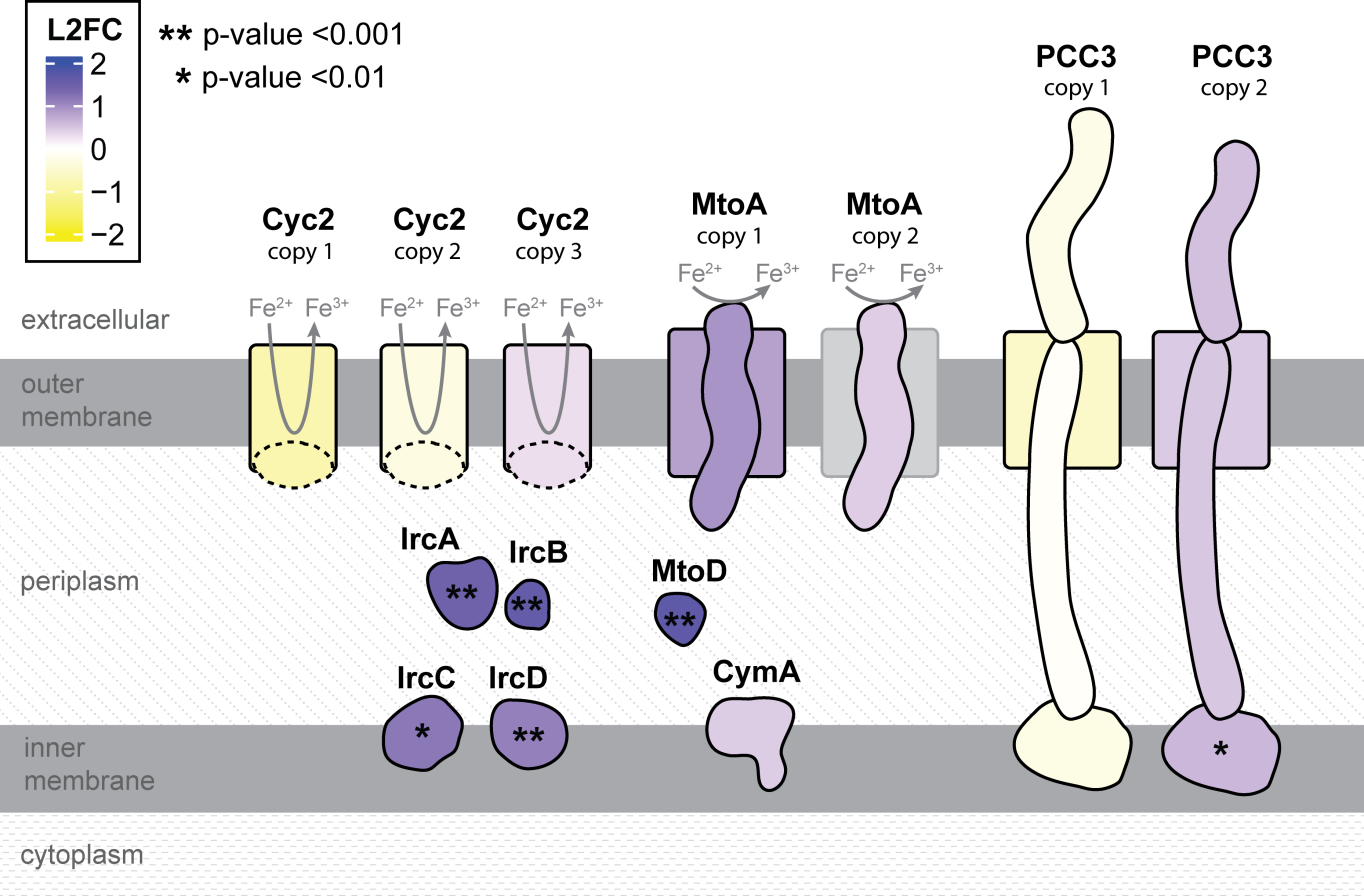
Cartoon schematic of iron oxidases, outer membrane cytochrome, porin, periplasmic, and inner membrane proteins colored by log2 fold change (L2FC) in normalized, rlog transformed gene expression between day 14 and day 6. Positive numbers (purple) were upregulated on day 14 relative to day 6. Asterisks indicate that the gene encoding a particular protein was significantly upregulated.

Both known iron oxidases, Cyc2 and MtoAB, are predicted to be outer membrane, porin-cytochromes, allowing them to oxidize Fe(II) outside the cell. Though functionally unverified, porin-cytochrome *c* complex 3 (PCC3) is predicted to be an outer membrane porin-cytochrome complex similar to, but larger than, the MtoAB iron oxidase complexes (24). PCC3 is widely distributed among various FeOB genomes (19, 24), making PCC3 another potential iron oxidation mechanism. *Sideroxydans* sp. CL21 has two copies of the PCC3 gene cluster. Each consists of four genes predicted to encode an extracellular multiheme cytochrome *c* (MHC), a porin, a periplasmic MHC, and an inner membrane protein. Analysis of the *Z*-scores, where mean expression is 0, showed both copies of PCC3 were expressed higher than the mean (*Z*-score ~ 1; Fig. S2), yet not as highly as *cyc2* or *mtoA* (*Z*-score ~ 3; Fig. 5A and B) across all three time points. From day 6 to day 14, PCC3 copy 1 was downregulated, while PCC3 copy 2 was upregulated, though the only significant (*P*-value < 0.01) change was to the inner membrane protein of PCC3 copy 2 (Fig. 6, Fig. S1).

Iron oxidases are just one part of FeOB extracellular electron transport pathways, which require additional cytochromes to convey electrons to the terminal oxidase or quinone pool. *Sideroxydans* sp. CL21 has genes encoding two *c*-type cytochromes, the monoheme MtoD and tetraheme CymA/ImoA, co-located in the genome next to *mtoA* copy 1 (Fig. 5D). Based on genomic context and previous genetic/biochemical characterization, these proteins may form part of the Mto iron oxidation pathway, moving electrons from the MtoA iron oxidase to the electron acceptor and/or quinone pool (30–33). Here, *mtoD* and *cymA/imoA* were upregulated at late log (day 14) relative to day 6, with *mtoD* being significantly upregulated (*P*-value < 0.001; Fig. 5 and 6). In this case, the iron oxidation capability corresponds best with expression of the periplasmic electron carrier gene *mtoD*.

*Sideroxydans* sp. CL21 also has homologs to the four “Fe(II)-responsive” genes identified in *S. lithotrophicus* ES-1 (Slit_1321-Slit_1324 [10]). We refer to these genes as “*ircABCD”* for “iron responsive cluster.” The *irc* genes encode two *c*-type cytochromes, diheme (IrcA) and monoheme (IrcB), both predicted to be periplasmic. They also encode a hypothetical protein (IrcC) and a *b*-type cytochrome (IrcD), with predicted localization in the inner membrane (Fig. 6). These genes were upregulated when *S. lithotrophicus* ES-1 was grown on Fe(II) vs thiosulfate (10) and were also highly expressed in *Sideroxydans* sp. CL21 when grown with Fe(0) (26). In our lactate experiment, expression of these genes increased as cells approached late-log phase (day 14), and all were significantly upregulated on day 14 relative to day 6 (Fig. 5C and 6). This significant upregulation corresponds to the cells’ ability to oxidize iron on day 14.

Overall, *Sideroxydans* sp. CL21 seems to be concurrently expressing genes for its known and putative iron oxidases (*cyc2*, *mtoA*, and PCC3) regardless of whether cells are capable of iron oxidation. However, the expression of genes for periplasmic and inner membrane proteins (*mtoD*, *ircABCD*, inner membrane component of PCC3 copy 2) varies. These genes were significantly upregulated in cells that showed iron oxidation activity, making them potential markers of iron oxidation activity.

## DISCUSSION

In this study, we demonstrated that Gallionellaceae *Sideroxydans* sp. CL21 exhibits remarkable metabolic versatility. *Sideroxydans* sp. CL21 can utilize a wide range of substrates, including short-chain organic acids such as lactate and pyruvate, as well as oxidize various soluble inorganic substrates, such as Fe(II) and thiosulfate. Additionally, it can metabolize mineral substrates, including magnetite and elemental sulfur [S(0)]. Overall, our findings show that *Sideroxydans* sp. CL21 is capable of organotrophy and lithoautotrophy, with the potential for mixotrophy and organoheterotrophy. This metabolic flexibility likely enhances its competitiveness and enables it to contribute to a wide range of biogeochemical transformations. Understanding the ecophysiology and biogeochemical roles of facultative iron oxidizers like *Sideroxydans* sp. CL21 requires more than just detecting their presence in environmental samples. We need a means to decipher whether these FeOB are actively oxidizing iron. Developing methods to directly track microbial iron oxidation activity requires (i) the identification of genes encoding the iron oxidation pathway and (ii) determining which of these genes can serve as reliable markers of microbial iron-oxidizing activity.

To be a marker of activity, a gene needs to increase in expression with iron oxidation and have minimal to no expression when cells are unable to oxidize iron. Validating marker genes requires study of facultative FeOB, of which there are few in culture, and ideally, there needs to be a condition under which cells *cannot* oxidize iron. Previous studies on *S. lithotrophicus* ES-1 showed differential gene expression of cells grown on Fe(II)-citrate and thiosulfate, but thiosulfate-grown cells were able to oxidize iron quickly with no lag after the substrate was switched to Fe(II). In contrast, *Sideroxydans* sp. CL21 grown on lactate was surprisingly unable to oxidize iron until late-log phase (day 14), giving an opportunity to compare gene expression of iron-oxidizing and non-iron-oxidizing conditions.

### Relationship between iron oxidation gene expression and activity

Although significant progress has been made in assigning function to the key iron oxidases such as Cyc2 and MtoA (10, 20, 33–35), the pathway is likely more complex, and additional iron oxidases may yet be discovered. Genes encoding the larger multiheme cytochrome complexes PCC3_1 and PCC3_2 are expressed at higher than mean levels. PCC3s are predicted to be large outer membrane cytochrome (OMC)-porin complexes (Fig. 6) (24), and while their function has not been tested, as OMCs, they could, in principle, oxidize iron. Thus, based on the *Sideroxydans* sp. CL21 transcriptome, there are at least four possible iron oxidases (Cyc2, MtoA, the PCC3_1, and PCC3_2 cytochromes) with various proteins that could direct electrons to terminal oxidases or reverse electron transport pathways.

One might expect the expression of iron oxidase genes to correlate with iron oxidation, but our results show that the well-characterized iron oxidase genes *cyc2* and *mtoA* are always highly expressed in our experiments and thus do not correspond to iron oxidation ability. This differs from *S. lithotrophicus* ES-1, which has very low expression of *mtoA* on dissolved Fe^2+^ but upregulates *mtoA* when grown on solid Fe(II)-containing minerals smectite and magnetite (10, 22, 23). We showed that *Sideroxydans* sp. CL21 grows on magnetite, and though magnetite was not present in lactate-grown cultures, expression of *mtoA* may still indicate a readiness to take up electrons from solid minerals. *Sideroxydans* sp. CL21 is more like *S. lithotrophicus* ES-1 in its high expression of *cyc2*. In *S. lithotrophicus* ES-1, expression of *cyc2* (one of three copies) is high even when cells are cultured on thiosulfate (10). Similarly, *Sideroxydans* sp. CL21 highly expresses two of its three *cyc2* copies when grown on lactate. Expression of *cyc2* is also generally high in marine iron-oxidizing Zetaproteobacteria (20). However, in *S. lithotrophicus* ES-1, additional copies of *cyc2* are iron-responsive (10). In theory, Cyc2 production could be post-transcriptionally regulated. However, this is not the case in *S. lithotrophicus* ES-1 since proteome data show that Cyc2 is highly expressed on Fe(II) (dissolved Fe^2+^ and magnetite [23]) and thiosulfate (25). One explanation for the overall high expression is that these FeOB maintain readiness or, in the case of *Sideroxydans* sp. CL21, partial readiness to oxidize iron. In any case, because *cyc2* expression does not correspond to iron oxidation activity, *cyc2* (alone) is not an accurate indicator of biotic iron oxidation.

Beyond iron oxidases, iron oxidation activity also requires electron carrier proteins in the periplasm and inner membrane. Currently, there is limited evidence supporting other essential components of the pathway, particularly periplasmic electron carriers and inner membrane proteins (Table S3). Our findings contribute to filling this gap by providing evidence for the involvement of genes encoding predicted periplasmic and inner membrane proteins. In fact, the genes that were most upregulated in cells that showed iron oxidation (day 14) were periplasmic *c*-type cytochrome genes *ircA*, *ircB*, and *mtoD* (Fig. 6), followed by the inner membrane protein genes *ircC*, *ircD*, and the PCC3_2 inner membrane component. There is accumulating evidence for the involvement of *irc* genes in iron oxidation. Homologs are widespread among Gallionellaceae and found in several other FeOB, including *Leptothrix cholodnii* SP-6, *Rhodoferax* spp., and several Zetaproteobacteria (Table S2) (19, 21). In *S. lithotrophicus* ES-1, *irc* genes are upregulated in iron-oxidizing cultures vs thiosulfate-oxidizing ones (10). The periplasmic di- and monoheme *c*-type cytochrome genes (*ircA* and *ircB*) are homologs of *grcJ* and *grcI* in *Ghiorsea bivoria* TAG-1. In *G. bivoria* TAG-1, GrcJ is significantly upregulated under iron-oxidizing vs H_2_-oxidizing conditions, and expression of *grcJ* increases over time when cells are grown with Fe(II) (21). In lactate-grown *Sideroxydans* sp. CL21, upregulated expression of *ircABCD* corresponded to the cells’ potential to oxidize iron in late-log cultures (day 14). Thus, it appears that cells increase expression of electron carrier genes like *irc*, *grc*, and *mtoD* when cells are ready to oxidize iron, making them the best candidates thus far for markers of iron oxidation potential.

The results to date suggest that the iron oxidation pathway is not regulated as a complete set in the neutrophilic FeOB Gallionellaceae. Instead, at least one OMC (one Cyc2) is constitutively expressed, which could serve multiple purposes. Cyc2 may operate as an Fe(II) sensor to tell the cell when it is time to increase expression of the iron oxidation pathway. An issue with this pathway is that OMCs are complicated to produce; they must be secreted to the periplasm, where the heme cofactor is attached, and then inserted into the outer membrane with proper folding. Each of these three steps involves separate machinery, so this requires substantial space in the inner membrane and periplasm, which may interfere with the electron transfer pathway. Thus, expressing Cyc2 and other OMCs ahead of iron oxidation may be a strategy for a quicker iron oxidation response. In contrast to the OMC, many of the periplasmic and inner membrane components are smaller and would be easier to produce when they are required. This strategy also allows the organism to express different periplasmic and inner membrane proteins to match different iron substrates [Fe(II), magnetite, and chelated Fe(II)], which have varied redox potentials (36, 37), as is the case in *Geobacter* (38).

Thus, we conclude that the Gallionellaceae may implement a multistage production of the iron oxidation pathway, with a subset of the OMCs expressed proactively, followed by additional OMCs and the rest of the pathway once iron oxidation is required. This may be further tested as additional facultative FeOB are isolated. In the meantime, we will need to carefully parse the expression profiles to interpret FeOB activity in the environment, though the *irc*/*grc* genes are promising markers.

### Carbon utilization among FeOB

*Sideroxydans* sp. CL21 is distinct among the current Gallionellaceae isolates in its ability to grow on organic acids. However, other uncultured mixotrophic or organo(hetero)trophic FeOB are likely present in organic-rich environments. A pangenomic analysis of Gallionellaceae isolates and environmental metagenome assembled genomes (MAGs) reveals that the potential for lactate utilization is rare but not unique, as it has been observed in some genomes from the Crystal Bog humic lake (Wisconsin) and freshwater lakes near Kuujjuarapik-Whapmagoostui (Canada) (19, 39–41). Active organic substrate utilization by FeOB fundamentally alters their role in the carbon cycle. While FeOB are traditionally considered autotrophs that fix CO_2_, the ability to grow organotrophically suggests that they may also contribute to the degradation of organic carbon *in situ*. The ability to grow on organics may provide a significant ecological advantage in specific habitats, especially wetlands and peatlands. In peatlands, mixotrophic FeOB face competition with both aerobic and anaerobic microorganisms for organic intermediates such as lactate and pyruvate. However, FeOB exhibits a unique advantage by coupling the consumption of these substrates to aerobic respiration under microaerophilic conditions. This metabolic flexibility of FeOB enables them to thrive in environments like peatlands, where fluctuating water tables create dynamic oxic-anoxic gradients, offering transient microaerophilic niches. Thus, such versatility may be key to their survival and dominance in specific ecosystems and play integral roles in biogeochemical iron and carbon cycling in dynamic environments.

### Implications of FeOB organotrophy for wetland carbon cycling

By demonstrating the versatility of FeOB metabolism and its genetic basis, our findings provide a foundation for understanding the broader biogeochemical roles of FeOB in wetland ecosystems and how they connect the iron and carbon cycles. It is already recognized that organotrophic iron-reducing bacteria can compete for organic carbon, thus shunting carbon away from methanotrophs, which could reduce wetland methane emissions (42–46). FeOB help promote this process by replenishing the supply of Fe(III). If the FeOB are autotrophic, then iron oxidation drives the regeneration of organic carbon, partly reversing the effect of organotrophic iron reduction and muting the effects on methanogenesis. But if the FeOB are organotrophic, they would join the competition for organics and, if sufficiently abundant and active, contribute to the suppression of methanogenesis. These are theoretical effects; evaluating the actual effects of FeOB organotrophy requires specifically monitoring FeOB carbon metabolism in the wetland environment and soil incubations. Potential iron oxidation gene markers would give us a way to link iron oxidation activity to organotrophic metabolisms via genome-resolved metatranscriptomics, paving the way toward deeper insights into FeOB influences on wetland biogeochemistry and interactions with wetland communities. This knowledge is essential for predicting how iron and carbon cycling—and associated greenhouse gas fluxes—will respond to environmental changes.

## MATERIALS AND METHODS

### Growth experiments

For all growth experiments, *Sideroxydans* sp. CL21 was cultured in triplicate, in 150 mL serum bottles with 80 mL of modified Wolfe’s minimal medium (containing 1.0 g NH_4_Cl, 0.5 g MgSO_4_⋅7H_2_O, 0.2 g CaCl_2_, and 0.05 g K_2_HPO_4_ per liter). All cultures were amended with a 0.1% (vol/vol) addition of each of ATCC trace vitamins and trace minerals. To keep micromolar concentrations of oxygen, headspaces were flushed daily with low-oxygen gas mix (2% O_2_, 20% CO_2_, and 76% N_2_). A Firesting oxygen spot sensor (Pyro Science) was used to measure and monitor oxygen concentrations. With the exception of lactate and pyruvate cultures, all bottles were incubated on their sides in a dark cabinet at room temperature. Lactate and pyruvate bottles were incubated upright in a New Brunswick Innova 42 shaking incubator at 20°C, 75 RPM. Growth of all cultures was measured by direct cell counting, in which cells were stained with Syto-13 and counted manually in a Petroff-Hauser counting chamber using fluorescence microscopy.

Lactate cultures included a one-time addition of sodium DL-lactate (CAS 72-17-3) for a concentration of 5 mM and were buffered with 10 mM of 2-(N-morpholino)ethanesulfonic acid (MES) at pH 6.0. Likewise, citrate (CAS 6132-04-3), pyruvate (CAS 113-24-6), thiosulfate (CAS 10102-17-7), and S(0) cultures were buffered with MES at pH 6.0 and given a one-time dose of sodium citrate dihydrate, sodium pyruvate, sodium thiosulfate pentahydrate, or elemental sulfur powder for a starting concentration of 5 mM. Biogenic and commercial magnetite cultures were each given a single addition of magnetite (1 g/L). Biogenic magnetite was synthesized by incubating *Shewanella oneidensis* MR-1 in Luria-Bertani media with two-line ferrihydrite for 10–12 days in an anaerobic chamber. After incubation, a magnet was used to hold the magnetite in place, while the supernatant was decanted. Minerals were washed 3× with deoxygenated water, then dried in an anaerobic desiccator.

FeCl_2_ cultures were buffered with 50 mM of MES at pH 6.0. These cultures were given a daily addition of FeCl_2_ ranging from 250 µM on day 0 to 500 µM on day 14. The FeCl_2_ + citrate experiment was conducted under these same conditions plus a one-time addition of 2.5 mM sodium citrate.

### Organic acid measurements

Samples from lactate and pyruvate cultures were collected, filtered with a 0.22 µm syringe filter, and stored at 4°C. The concentration of organic acids (lactate and pyruvate) was measured by high-performance liquid chromatography (HPLC). Standards for formate and acetate were also included to check for partial oxidation products, but neither was detected. Measurements were made on a Shimadzu HPLC with a Photodiode Array Detector (model MPD-40) with an Alltech Prevail Organic Acid 5 µm 150 × 4.6 mM column and guard column. Organic acids were eluted at room temperature with 25 mM KH_2_PO_4_ at pH 2.5, adjusted with phosphoric acid, at 1 mL/minute. Absorbance was read at 210 nm to quantify compounds based on peak area. Instrument control and data collection were performed using LabSolutions software (v. 5.106 SP1). Peak areas were exported to Microsoft Excel, where calibration curves were generated by linear regression of authentic standard peak areas and used to calculate concentrations.

### Transcriptome experiment

*Sideroxydans sp*. CL21 was cultured in 5 mM lactate as described above. Bottles were inoculated with stationary-phase, lactate-adapted cells. Cell counts and lactate measurements were taken on days 0, 3, 6, 9, and 14. On days 6, 9, and 14, aliquots of cultures were used in Fe(II) spike assays (described below), and bottles were sacrificed in biological sextuplets for transcriptome sequencing. For all sacrifices, a 1:10 (vol/vol) of stop solution (buffer-saturated phenol:absolute ethanol at a 1:9 [vol/vol]) was injected into the bottles to stop transcription. Cells were then harvested by filtering onto a 0.22 µm polyethersulfone (PES) filter and stored at −80°C until RNA extraction.

### Fe(II) spike assay

Fe(II) spike assays were performed at different time points to test *Sideroxydans* sp. CL21’s response to Fe(II) when grown on lactate and pyruvate. For these assays, 10 mL of culture was extracted from each of the six inoculated serum bottles. Of this, 5 mL was incubated in 5 mM sodium azide for 20 minutes to create the azide-killed control. The other 5 mL was untreated and served as the “live cell” samples. A 5 mL aliquot was taken from six uninoculated bottles for the uninoculated control. All samples were bubbled with a 2% O_2_, 20% CO_2_, and 78% N_2_ gas mix throughout the experiment to keep oxygen levels consistent and low. Each sample, live and control, was spiked with 20 µL of 100 mM FeCl_2_ for a 400 µM starting concentration. Samples were collected at 0, 5, 15, 30, and 60 minutes and preserved in a 1:1 mix with 40 mM sulfamic acid. After all samples were collected, a spectrophotometric ferrozine assay was used to measure ferrous iron concentrations (47, 48).

### RNA extraction and sequencing

RNA was extracted from cells collected on PES filters using a RNeasy PowerSoil RNA kit (Qiagen). Extracted RNA was treated with three RNase inhibitors (1 µL Invitrogen SUPERase, 3 µL RNaseOUT, and 1.25 µL Ambion RNase Inhibitor) to prevent degradation. Total RNA was quantified using a Qubit RNA HS assay kit from Invitrogen, and quality was assessed with an Agilent fragment analyzer. A Zymo RNA Clean and Concentrator−5 kit was used to remove excess salts and DNA from all samples. After clean-up, three samples with the best yield and quality from each timepoint (day 6: 12.6, 13.2, and 29.6 ng/µL; day 9: 34.6, 43.0, and 25.6 ng/µL; day 14: 82.0, 20.0, and 92.0 ng/µL), along with sample 6A from day 9 (33.4 ng/µL), were selected for sequencing. Ribosomal RNA depletion and library preparation were done using a Zymo-Seq RiboFree Total RNA Library Kit. Sixty-four nanograms of total RNA from each sample was sequenced on an Illumina NextSeq 2000 using a P2 100-cycle protocol, yielding up to 40 GB of data (up to 400 million single-end reads). All clean-up, library prep, and sequencing were performed by the University of Delaware DNA Sequencing and Genotyping Center.

### Transcriptome QC and analysis

Raw transcriptome reads were quality checked using FastQC v0.11.9 (49). Reads were trimmed, and adaptor sequences were removed using TrimGalore! v.0.6.6 (50) and Cutadapt v4.4 (51). A minimum quality score of 28 and a minimum length of 75 bp were applied to filter out poor reads. Post-trim, the read quality was reassessed with FastQC v0.11.9 (49), and SortMeRNA v4.3.6 (52) was run on all samples to remove any rRNA sequences before mapping.

Reads were mapped to the closed, RefSeq-annotated genome of *Sideroxydans* sp. CL21 (GCF_902459525.1) using Bowtie 2 v2.5.1 (53) and Samtools v1.10 (54). The average alignment rate per sample was 97%, with the exception of day 6, sample 3B, which was 73%. Read counts were reported using Htseq v2.0.2 (55). After comparing the total number of reads mapped for each sample and the overall alignment rates, we determined that day 6, sample 3B was an outlier with potential contamination and too few quality reads to be useful. To be thorough, we ran and compared the differential gene expression (DGE) analysis (described below) with and without the day 6 sample 3B. Exclusion of day 6 sample 3B did not affect the overall findings or change whether the differential expression of our genes of interest was significant. Instead, removing the outlier decreased noise and made the data set more precise.

Differential gene expression was analyzed in R v4.4.0 (56) with RStudio v2023.06.0 Build 421 (57) using the DESeq2 v1.26.0 (58) package. Data were normalized using DESeq's default algorithm, which uses an estimation of size factors to control for differences in sequencing depth. *P*-values were determined with the Wald Test and adjusted for false discovery rate using the Benjamini-Hochberg correction. An alpha of 0.01 was set as the cut-off for significance. Reports of normalized count data for each sample were generated using the regularized logarithm (rlog) transformation (58). These rlog count data were used to calculate *Z*-score to analyze the variance of individual genes across samples. To test the validity of these transformations, we checked the expression of housekeeping genes for DNA gyrase and RNA polymerase (*gyrA, gyrB*, and *rpoA*). Their even expression over all time points indicates that the rlog and *Z*-score transformations were successful (Fig. S2A; Table S1).

### Identification and analysis of the Fe(II)-responsive (*irc*) gene cluster

Homologs of the Fe(II)-responsive (*irc*) gene cluster were identified by using cblaster (59) to search the NCBI database for BLAST hits (*e*-value cut-off <1E-10) to the *irc* genes of *Sideroxydans* sp. CL21 that were located within 20,000 bp of each other in their respective genomes. Positive hits needed to contain at least three of the genes, excluding the Hsp33 chaperone. Results were visualized using Clinker (60). PSORTb 3.0 (61), Gneg-mPLoc (62), PredictProtein (63), and InterProScan (64, 65) were used to predict subcellular localization and domains of the *irc* genes.

## Data Availability

Transcriptomic data were uploaded to NCBI BioProject PRJNA1216980.

## SUPPLEMENTAL MATERIAL

Supplemental material aem.00395-25-s0001.pdf Figures S1 and S2 and Table S3.

Table S1 aem.00395-25-s0002.xlsx Differential expression, normalized expression, and Z-score data for key genes.

Table S2 aem.00395-25-s0003.xlsx *ircABCD* homologs in FeOB and FeRB.

ASM does not own the copyrights to Supplemental Material that may be linked to, or accessed through, an article. The authors have granted ASM a non-exclusive, world-wide license to publish the Supplemental Material files. Please contact the corresponding author directly for reuse.
